# A Peculiar Accessory Scrotum With a Human Tail‐Like Structure: A Case Report

**DOI:** 10.1155/crip/9623598

**Published:** 2026-07-27

**Authors:** Hiroshi Sonobe, Rika Omote, Ryutaro Kondo, Souji Ibuka, Ryuta Saka, Shun Iwasaki, Taku Yamamichi

**Affiliations:** ^1^ Department of Diagnostic Pathology, National Hospital Organization (NHO) Fukuyama Medical Center, Hiroshima, Japan; ^2^ Department of Gastroenterological Surgery/Pediatric Surgery, Gifu University Graduate School of Medicine, Gifu, Japan, gifu-u.ac.jp; ^3^ Department of Pediatric Surgery, Okinawa Prefectural Nanbu Medical Center and Children′s Medical Center, Okinawa, Japan; ^4^ Department of Pediatric Surgery, National Hospital Organization (NHO) Fukuyama Medical Center, Hiroshima, Japan

**Keywords:** case report, human tail, ischiorectal fossa, perianal accessory scrotum, tail bud

## Abstract

Accessory scrotum is a rare congenital anomaly resulting from aberrant scrotal development. It is usually present at birth in the perineum of male infants and is often associated with a lipoma at its base. Here, we report a peculiar case of an accessory scrotum present at birth on the skin adjacent to the anus. The accessory scrotum was continuous with a narrow stalk consisting of longitudinally oriented connective tissue and nerve bundles within the fatty tissue, which further extended to a terminal nodule containing articular cartilage and marrow tissues in the ischiorectal fossa, forming a 7‐cm‐long structure. The components of the stalk and terminal nodule resembled those described in the human tail but ran subcutaneously without a skin covering. Therefore, the lesion was diagnosed as an accessory scrotum with a human tail‐like structure. The lesion was completely resected at 10 months of age. Preoperative whole‐body computed tomography revealed no other anomalies, and the infant demonstrated normal development approximately 1 year postoperatively. To our knowledge, no similar cases have been reported, and its histogenesis remains unexplained. We postulated that this lesion may result from the ectopic migration of pluripotent cells derived from the tail bud during early embryonic development. This case provides unique insights into the shared embryological mechanisms between accessory scrotum and human tail‐like anomalies.

## 1. Introduction

Accessory scrotum is a rare anomaly that arises during fetal scrotal development [1]. It occurs in male infants, usually in the perineum, and is often associated with a lipoma at its base [2–9]. Surgical excision is the standard treatment. Recently, we encountered a unique case of an accessory scrotum located on the skin adjacent to the anus. This lesion was continuous with a narrow stalk containing longitudinally oriented nerve bundles and connective tissue within fatty stroma and extended to a terminal nodule composed of articular cartilage and bone marrow tissues in the ischiorectal fossa. To the best of our knowledge, no similar cases of an accessory scrotum involving musculoskeletal components have been reported. Here, we provide a comprehensive pathological description of this lesion and discuss the underlying embryological mechanisms.

## 2. Case Presentation

**Figure 1:**
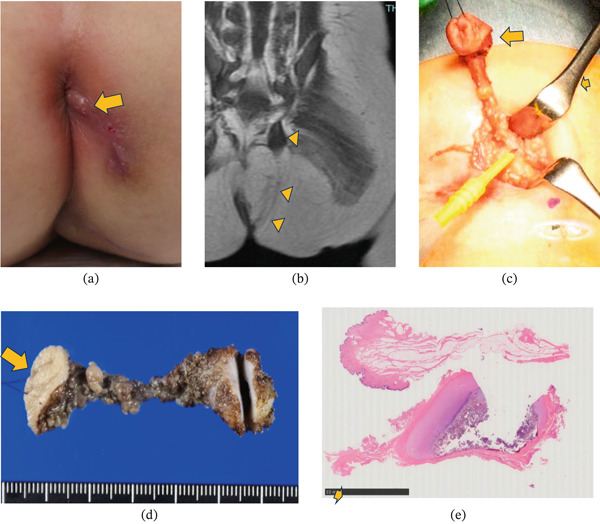
Clinical, radiological, and macroscopic findings of the lesion. (a) Preoperative view of the small, raised skin nodule located adjacent to the 5 o′clock position of the anus. (b) Coronal unenhanced T1‐weighted in‐phase pelvic magnetic resonance imaging (MRI) demonstrates a narrow cord‐like structure (lower arrowheads) extending from the perianal accessory scrotum through the perineal soft tissue to a distal nodular component (upper arrowhead) in the ischiorectal fossa. (c) Intraoperative view displaying the mobilization and resection of the skin nodule and stalk, with the firm nodule still remaining in the deeper tissue. (d, e) Macroscopic views of the excised, 7‐cm‐long dumbbell‐shaped specimen, comprising a 2‐cm perianal skin nodule, a soft slender stalk (4 mm thick, 4 cm long), and a firm distal nodule (2 cm in diameter).

The patient was a 10‐month‐old Japanese boy with an unremarkable family history. His parents had been aware of a small, raised perianal skin nodule since birth. At 10 months of age, he was referred to the Department of Pediatric Surgery at our institution. Physical examination revealed a skin nodule located adjacent to the 5 o′clock position of the anus, leading to a clinical diagnosis of an accessory scrotum (Figure 1a). Preoperative coronal unenhanced T1‐weighted in‐phase pelvic magnetic resonance imaging (MRI) revealed a continuous, narrow, cord‐like structure extending from the perianal accessory scrotum, through the perineal soft tissue, to a calcified nodular component within the ischiorectal fossa. This imaging finding detailed the anatomical path of the accessory scrotum component as confirmed by the radiological investigation (Figure 1b). Systemic screening showed no other anomalies.

The entire lesion was successfully resected (Figure 1c). Macroscopically, the excised dumbbell‐shaped lesion measured approximately 7 cm in total length. It comprised a 2‐cm skin nodule near the anus, a soft slender stalk measuring 4 mm in thickness and 4 cm in length, and a firm nodule 2 cm in diameter (Figure 1d,e). The specimen was fixed in 10% buffered formalin and sectioned longitudinally. Tissue sections were cut at a thickness of 4 *μ*m and stained with hematoxylin and eosin (H&E) for routine histological examination. Additionally, immunohistochemical analysis was performed using a Ventana BenchMark ULTRA automated stainer with ready‐to‐use primary antibodies against *α*‐smooth muscle actin (*α*‐SMA) and S‐100 protein.

**Figure 2:**
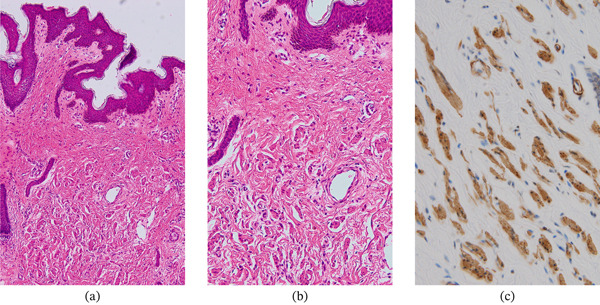
Histopathological findings of the perianal skin lesion. (a) Low‐power view demonstrating prominent epidermal folds (H&E, 40×). (b) Medium‐power view showing numerous thick smooth muscle bundles clustered within the dermis (H&E, 200×). (c) Immunohistochemical staining demonstrating strong immunoreactivity for alpha‐smooth muscle actin (*α*‐SMA) within the dermal smooth muscle bundles (400×).

**Figure 3:**
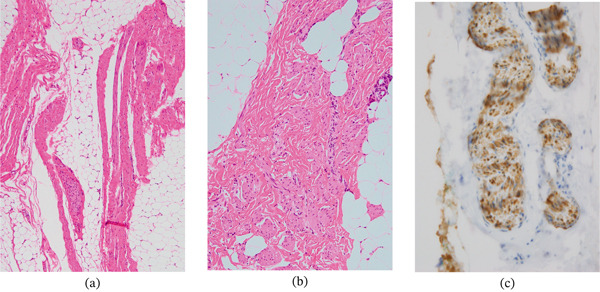
Histopathological features of the intermediate narrow stalk of the lesion. (a) Low‐power view demonstrating that the stalk is composed of longitudinally arranged bundles of fibroconnective and peripheral nerve tissues embedded within an adipose stroma (H&E, 40×). (b) Higher magnification of (a), highlighting the detailed architecture of the fibroconnective and neural components (H&E, 200×). (c) Automated immunohistochemical staining showing clear positivity for S‐100 protein within the peripheral nerve bundles (400×).

**Figure 4:**
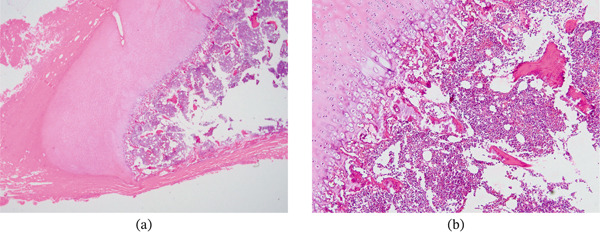
Histopathological features of the hard nodule at the distal end of the lesion. (a) Low‐power view displaying the overall architecture of the articular cartilage and underlying bone marrow tissue (H&E, 40×). (b) Higher magnification highlighting the detailed cellular structures of the cartilage and marrow elements (H&E, 100×).

Histologically, the perianal skin lesion demonstrated features consistent with an accessory scrotum, characterized by prominent epidermal folds and numerous smooth muscle bundles within the dermis (Figure 2a–c). A narrow, soft stalk extending 4 cm from the accessory scrotum to the firm distal nodule was composed of longitudinally arranged fibroconnective and peripheral nerve tissue bundles embedded within an adipose stroma (Figure 3a–c). The firm nodule itself consisted of mature articular cartilage and underlying bone marrow tissue (Figure 4a,b). However, the stalk was not covered by skin.

Based on these findings, which demonstrated a unique combination of an accessory scrotum and human tail‐like components, the definitive pathological diagnosis was established as an accessory scrotum with a human tail‐like structure. At the 1‐year postoperative follow‐up, the patient remains healthy with no adverse events or recurrence.

## 3. Discussion

### 3.1. Embryological and Histological Characteristics of Accessory Scrotum

The genital tubercle and labioscrotal folds develop from the cloacal membrane, which is common to both sexes, at approximately 4 weeks of fetal development. At approximately 15 weeks in females, the clitoris develops from the genital tubercle, and the labia majora develops from the labioscrotal folds. In males, the penis develops from the genital tubercle, and the scrotum develops from the labioscrotal folds, owing to the effects of male hormones secreted from the testes. Accessory scrotum is thought to arise from abnormalities in this scrotal formation process [1]. Histologically, a normal scrotum is characterized by epidermal folds and a tunica dartos composed of numerous smooth muscle bundles within the dermis. These characteristic features are also present in accessory scrotum lesions, providing the basis for histological diagnosis [1, 5]. In the present case, the skin lesion adjacent to the anus demonstrated epidermal folds and a tunica dartos, supporting the histological diagnosis of an accessory scrotum.

An accessory scrotum is a congenital perineal malformation in male infants, which is frequently associated with a lipoma at its base. It is estimated that approximately 70%–80% of these cases are accompanied by a perineal lipoma [2–9]. It usually presents as a peduncular or bead‐like mass at the base, and no testes are found within it. Moreover, in some cases, it is accompanied by various anomalies [10–12]. Here, in this case, a typical accessory scrotum was present at birth in a male infant, but unusual location, adjacent to the anus. From the accessory scrotum, a narrow stalk composed of adipose tissue, connective tissue, and nerve bundles, as well as a nodule composed of articular cartilage and bone marrow tissue, extended to the ischiorectal fossa. This structure extending from the accessory scrotum comprises components similar to those of the human tail. Nonetheless, because the present lesion is located in deep soft tissue, and is not covered by skin, we consider the term “human tail‐like structure” more appropriate.

### 3.2. Human Tail Classification and Application to the Present Case

During the 5th–6th week of fetal development, a caudal filament consisting of 10–12 tail bones, connective tissue, nerve tissue, vascular tissue, striated muscle tissue, and the overlying skin is formed. Around the 8th week, it typically degenerates and disappears; therefore, humans are normally born without a tail [13]. Human tails may result from the abnormal development and incomplete degeneration of this embryonic tail [14]. They appear as cord‐like or nodular protrusions in the sacrococcygeal region, comprising tissues such as fatty tissue, connective tissue, and nerve bundles covered by normal skin [15–18], and can be accompanied by malformations of the sacrum and spine [19–23].

Several classifications of the human tail have been proposed, although no consensus has been established. In the classic classification by Harrison [24], a caudal appendage is referred to as a “human tail in a broad sense” when a coccygeal component is present and as a “human tail in the narrow sense” when it is absent. According to the classification by Dao et al. [25], a “true tail” is composed of fat and connective tissue covered by normal skin and may contain striated muscle, blood vessels, and nerve components but lacks bone, cartilage, or a notochord. Conversely, lesions containing bone, cartilage, notochordal components, lipoma, or teratoma are termed “pseudotails” and are distinguished from true tails. These differences may reflect variability in the interpretation of abnormalities in embryonic tail development and regression. Moreover, another classification has been proposed based on whether human tails are associated with malformations of the sacrum or vertebrae [14].

The deep lesion continuous with the accessory scrotum in this case consisted of adipose tissue, connective tissue, nerve components, articular cartilage, and bone marrow components. Except for the lack of an independent skin coverage, it histologically represents the components of a “pseudotail” as classified by Dao et al. [25].

Ibuka et al. [9] reported a dumbbell‐shaped protruding skin lesion adjacent to the dorsal site of the anus at birth in a male infant. They found that the skin lesion was an accessory scrotum with a thin stalk extending from it. Connective tissue, nerves, and vascular components ran longitudinally within the fatty tissue, and the basal nodule consisted of a lipoma. The entire lesion, including the stalk and lipoma, was completely covered by skin structures, suggesting that it comprised an accessory scrotum, a human tail‐like structure, and a lipoma.

In distinct contrast to their case, where the tail‐like structure was completely subcutaneous and covered by its own skin, the deep, tail‐like structure in the present case extended into the ischiorectal fossa and lacked a cutaneous covering. To our knowledge, this is the first reported case of an accessory scrotum associated with such a deep, non–skin‐covered human tail‐like structure. Therefore, due to these unique anatomical characteristics, explaining the histogenesis of the present lesion based solely on existing literature remains challenging.

### 3.3. Differential Diagnosis of the Present Case

Regardless of the presence of an accessory scrotum, benign masses arising in the perineum or pelvic soft tissues—such as hamartomas, choristomas, lipomas, dermoid cysts, and teratomas—must be differentiated from the present lesion [26]. A hamartoma is characterized by an abnormal proliferation of mature tissues indigenous to the site, distinguishing it from a choristoma (heterotopic rest) consisting of normal tissue in an abnormal location. A lipoma is composed of mature adipocytes, whereas a dermoid cyst is lined by stratified squamous epithelium without atypia, rarely undergoing malignant transformation [27, 28]. Teratomas are true tumors containing tissues derived from all three germ layers and are classified as mature or immature [29]. Although the sacrococcygeal region is a common site for these benign lesions, the human tail‐like structure in this case did not meet the diagnostic criteria for any of these categories.

### 3.4. Embryological Development of the Present Lesion

Histologically, the deep lesion continuous with the accessory scrotum was composed of bone, cartilage, adipose, and neural tissues. Regarding its embryological development, the following hypotheses may be proposed, although they remain speculative. Normally, a cluster of cells known as the “tail bud,” which appears between gestational weeks 5 and 6, possesses the pluripotency to differentiate into various tissue lineages [30]. As the embryo grows, however, these cells become redundant and typically undergo programmed cell death (apoptosis) by the 8th week of gestation [31].

One hypothesis for the present case is that an abnormality in intercellular signaling [32] might have caused some of these tail bud cells, which should have regressed, to persist within the ischiorectal fossa. This potentially led to continued growth and differentiation in that region, resulting in a structure resembling a “human tail.” Alternatively, it is conceivable that during secondary neurulation (the process of spinal cord formation) [33, 34], these pluripotent cells aberrantly migrated to the perineal site and differentiated into various musculoskeletal and neural tissues. While such aberrant migrations are generally reported to be accompanied by spinal and spinal cord malformations [35], no such abnormalities were observed in our patient.

In conclusion, we described a peculiar case of an accessory scrotum located in the skin adjacent to the anus. The accessory scrotum extended to a narrow stalk composed of nerve bundles, connective tissue, and adipose tissue as well as a nodule consisting of articular cartilage and bone marrow tissues that was embedded deep within the soft tissue. Because these features resemble those of the human tail, we report this lesion as an accessory scrotum with a human tail‐like structure. Since no similar cases of accessory scrotum have been reported to date, we reported here the detailed pathological descriptions of this lesion and discussed its embryological pathogenesis. Further accumulation of similar cases is warranted to better understand the clinical behavior and syndromic associations of this rare anomaly.

## Author Contributions

Hiroshi Sonobe contributed to the conceptualization, methodology, data collection, and writing and editing of this manuscript. Hiroshi Sonobe, Rika Omote, and Ryutaro Kondo discussed the histological findings and diagnosed the present case. Souji Ibuka, Ryuta Saka, Shun Iwasaki, and Taku Yamamichi performed surgery and follow‐up studies in the present case.

## Funding

No funding was received for this manuscript.

## Ethics Statement

Signed informed consent for the participation and publication of medical details was obtained from the parents/guardians of the male infant. The authors declare that ethics committee approval was not required for this case report.

## Conflicts of Interest

The authors declare no conflicts of interest.

## Data Availability

Some data are available upon request due to ethical considerations. Data will be shared in accordance with the institutional guidelines after obtaining necessary permissions.
